# Supplementary material for: Genome-resolved metagenomics suggests a mutualistic relationship between Mycoplasma and salmonid hosts
Source: Commun Biol. 2021 May 14;4:579. doi: 10.1038/s42003-021-02105-1 (PMC8121932; doi:10.1038/s42003-021-02105-1)
Supplement: Supplementary file 2 — Supplementary Information [file 42003_2021_2105_MOESM2_ESM.pdf]

Supplementary Information

Title: “Genome-resolved metagenomics suggests a mutualistic relationship between *Mycoplasma* and salmonid hosts”

Authors:
Jacob Agerbo Rasmussen (1,2)*
Kasper Rømer Villumsen (3)
David A. Duchêne (2)
Lara Christine Puetz (2)
Tom O. Delmont (2,4)
Harald Sveier (5)
Louise von Gersdorff Jørgensen (6)
Kim Præbel (7)
Michael D. Martin (8)
Anders Miki Bojesen (3)
M. Thomas P. Gilbert (2,7)
Karsten Kristiansen (1,9)
Morten Tønsberg Limborg (1,2)*

Asterisks indicate corresponding authors and their contact information are listed below:
Jacob Agerbo Rasmussen: jacob.rasmussen@bio.ku.dk
Morten Tønsberg Limborg: morten.limborg@sund.ku.dk

Author information:
1) Laboratory of Genomics and Molecular Medicine, Department of Biology, University of Copenhagen, Copenhagen, Denmark
2) Center for Evolutionary Hologenomics, GLOBE institute, Faculty of Health and Medical Sciences, University of Copenhagen, Copenhagen, Denmark
3) Department of Veterinary and Animal Sciences, University of Copenhagen, Veterinary Clinical Microbiology, Copenhagen, Denmark
4) Génomique Métabolique, Genoscope, Institut François Jacob, CEA, CNRS, Univ Evry, Université Paris-Saclay, 91057 Evry, France
5) Lerøy Seafood Group ASA, N-5020 Bergen, Norway
6) Department of Veterinary and Animal Sciences, University of Copenhagen, Parasitology and Aquatic Pathobiology, Copenhagen, Denmark
7) Norwegian College of Fishery Science, UiT the Arctic University of Norway, N-9037 Tromsø, Norway
8) Department of Natural History, NTNU University Museum, Norwegian University of Science and Technology (NTNU), Trondheim, Norway
9) Institute of Metagenomics, BGI- Shenzhen, Shenzhen, China

Orcid IDs:
Jacob Agerbo Rasmussen https://orcid.org/0000-0002-7710-8912
Kasper Rømer Villumsen https://orcid.org/0000-0002-0616-4095
David A. Duchêne https://orcid.org/0000-0002-5479-1974
Lara Christine Puetz https://orcid.org/0000-0002-0323-799X
Tom O. Delmont https://orcid.org/0000-0001-7053-7848
Harald Sveier https://orcid.org/0000-0001-6907-5340
Kim Præbel https://orcid.org/0000-0002-0681-1854
Louise von Gersdorff Jørgensen https://orcid.org/0000-0002-6005-3249
Michael D. Martin http://orcid.org/0000-0002-2010-5139
Anders Miki Bojesen https://orcid.org/0000-0003-4030-0019
M. Thomas P. Gilbert https://orcid.org/0000-0002-5805-7195
Karsten Kristiansen https://orcid.org/0000-0002-6024-0917
Morten Tønsberg Limborg https://orcid.org/0000-0002-7718-6531

Bacterial biomass monitoring in rainbow trout

Supplementary Table 1) Real time PCR (qPCR) monitoring of bacterial 16S rRNA gene in intestinal samples of rainbow trout

Sample	Dilution	CT value	Group Average	Standard Deviation (SD)
Extraction Neg	1:1	40.0	38.5	2.1
Extraction Neg	1:1	37.1	38.5	2.1
RT1	1:1	33.6	36.3	2.8
RT1	1:10	34.1	36.3	2.8
RT2	1:1	40.0	36.3	2.8
RT3	1:1	34.4	36.3	2.8
RT3	1:10	34.9	36.3	2.8
RT5	1:1	40.0	36.3	2.8
RT5	1:10	37.3	36.3	2.8
PCR.Blk	1:1	39.8	39.9	0.1
PCR.Blk	1:1	40	39.9	0.1
Water sample 1	1:1	26.99	27.6	2.8
Water sample 2	1:1	25.12	27.6	2.8
Water sample 3	1:1	30.68	27.6	2.8

Host genomes for host filtering

Supplementary Table 2) Host genomes for host filtering

Species	NCBI Taxonomy ID	Accesion ID
Oncorhynchus mykiss	NCBI:txid8022	GCF_002163495.1_Omyk_1.0
Salmo salar	NCBI:txid8030	GCF_000233375.1_ICSASG_v2
Coregonus sp.	NCBI:txid861768	GCA_902175075.1_AWG_v1

Data Generation and Quality Control

Supplementary Table 3) Shotgun sequencing output and quality control output

Sample	Host Species	No. Million Raw Reads	Paired End Length (bp)	No. Million Reads post QC & Filtering
RT1_1	Oncorhynchus mykiss	216.424	150	26.061
RT1_2	Oncorhynchus mykiss	216.424	150	31.428
RT2_1	Oncorhynchus mykiss	210.547	150	16.691
RT2_2	Oncorhynchus mykiss	210.547	150	20.406
RT3_1	Oncorhynchus mykiss	195.024	150	10.318
RT3_2	Oncorhynchus mykiss	195.024	150	12.167
RT4_1	Oncorhynchus mykiss	227.968	150	8.168
RT4_2	Oncorhynchus mykiss	227.968	150	10.838
RT5_1	Oncorhynchus mykiss	209.142	150	15.078
RT5_2	Oncorhynchus mykiss	209.142	150	16.206
AS1_1	Salmo salar	46.733	100	0.885
AS1_2	Salmo salar	46.733	100	0.885
AS3_1	Salmo salar	24.024	100	4.349
AS3_2	Salmo salar	24.024	100	4.349
AS4_1	Salmo salar	13.521	100	15.871
AS4_2	Salmo salar	13.521	100	15.871

AS5_1	Salmo salar	53.497	100	4.448
AS5_2	Salmo salar	53.497	100	4.448
AS6_1	Salmo salar	32.376	100	6.139
AS6_2	Salmo salar	32.376	100	6.139
AS7_1	Salmo salar	45.849	100	1.951
AS7_2	Salmo salar	45.849	100	1.951
AS142_1	Salmo salar	53.735	100	6.778
AS142_2	Salmo salar	53.735	100	6.778
AS391_1	Salmo salar	62.770	100	2.588
AS391_2	Salmo salar	62.770	100	2.588
WF_1	Coregonus lavaretus	46.429	100	21.754
WF_2	Coregonus lavaretus	47.398	100	22.218

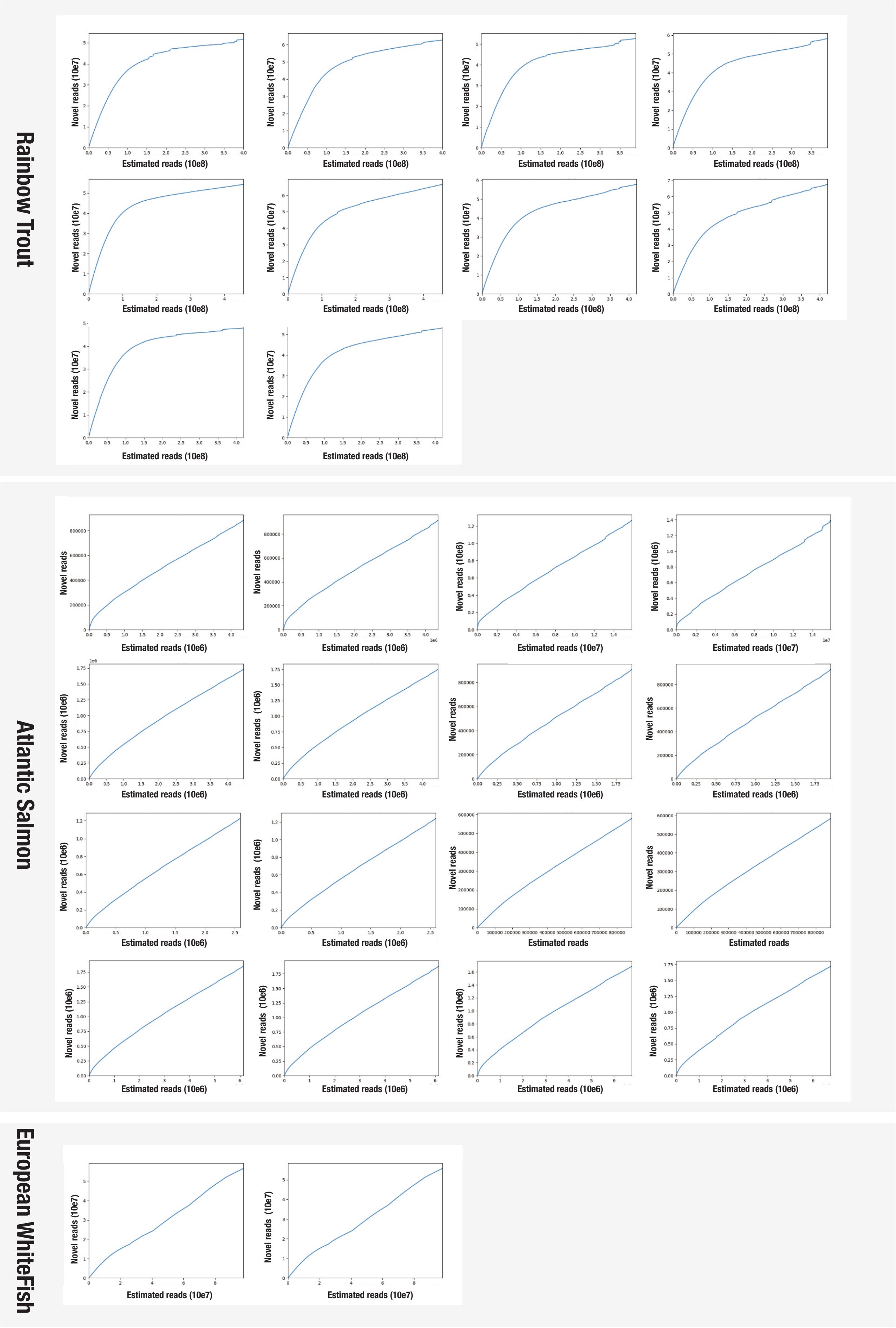
Rainbow Trout
Atlantic Salmon
European WhiteFish
Novel reads (10e7)
Estimated reads (10e8)
Novel reads
Estimated reads (10e6)
Novel reads (10e6)
Estimated reads (10e7)
Estimated reads

Supplementary Figure 1. Collector's curves of all paired end reads for rainbow trout, Atlantic salmon, and European whitefish, estimated using khmer. Saturation of curves indicate saturated read depth for 5X of all reads.

Saturation of read depth for 5X coverage is only fully saturated for rainbow trout. Atlantic salmon and European whitefish would need more data for fully saturated read depth, as seen for a nearly linear progression between accumulated data versus novel reads found. Taken the completion of *Candidatus Mycoplasma lavaretus* (>99% completion) and *Candidatus Mycoplasma salmoninae salar* (>85% completion) compared to *Candidatus salmoninae mykiss* (>95% completion)*,* the lack of read depth is not expected to affect the investigation of dominant species, like *Mycoplasma*, in the metagenomes of Atlantic salmon and European whitefish.

Quality Control of Assembled *Mycoplasma* MAGs

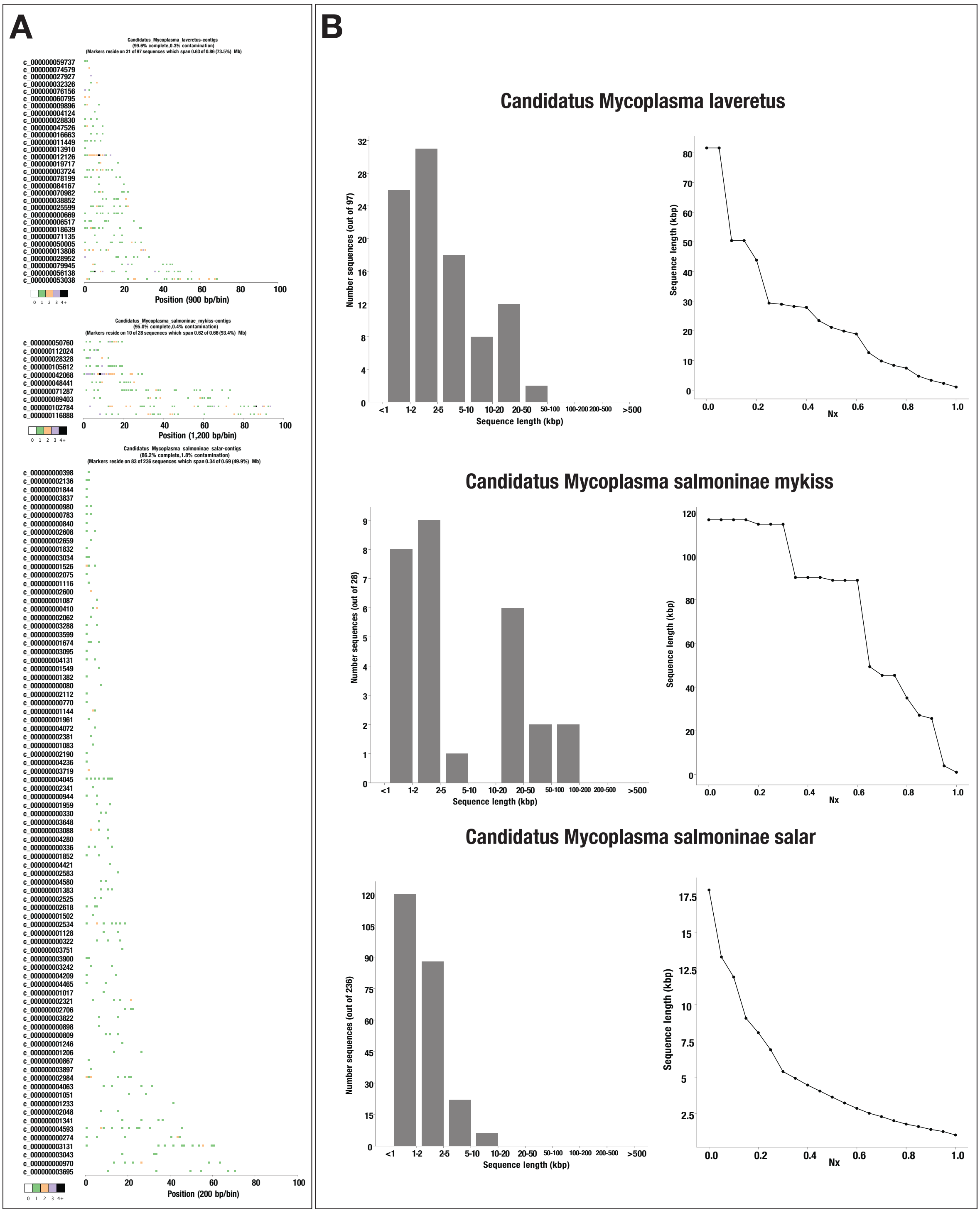

Supplementary Figure 2. Quality control of metagenomic assembled genomes (MAGs) from European whitefish (*Candidatus* Mycoplasma laveretus), rainbow trout (*Candidatus* Mycoplasma salmoninae mykiss), and Atlantic salmon (*Candidatus* Mycoplasma salmoninae salar), using CheckM. A) Hidden Markov Model (HMM) hits across assembled contigs for each *Mycoplasma* related MAG, providing information regarding the extent to which marker genes are collected. The number of marker genes within a fixed size window is indicated by different colours. Sequences without any marker genes are not shown. B) Histogram of contig length and line chart of ratio of MAG described by a given sequencing length for each *Mycoplasma* related MAG.

Comparison of novel MAGs from each host individual from Atlantic Salmon, Rainbow Trout, Common Whitefish

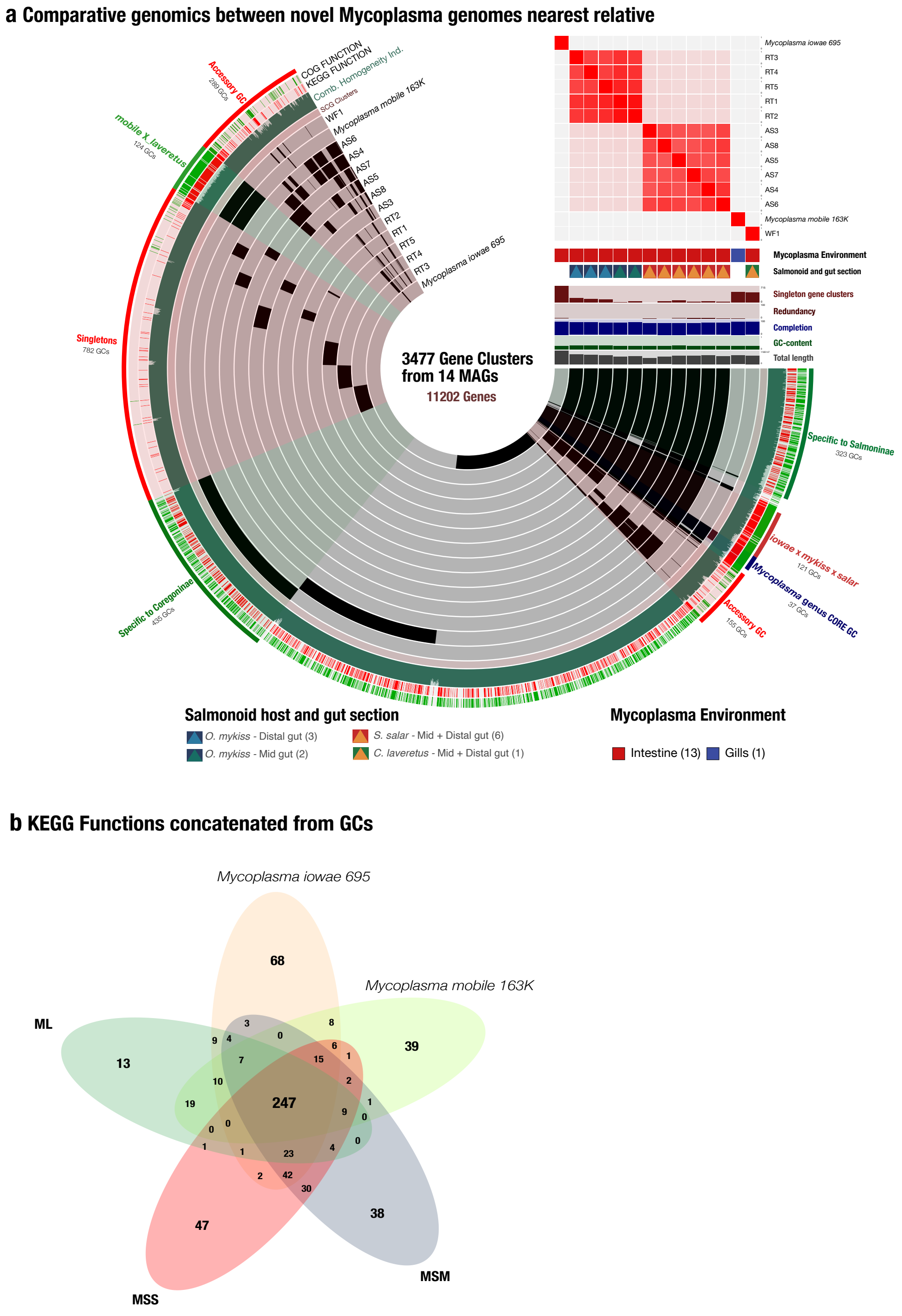

Supplementary Figure 3. Comparison of novel *Mycoplasma* with nearest relatives across sampled individuals. A) Comparative genomics between the novel *Mycoplasma* single assembled MAGs. Each one of the 3477 gene clusters (GCs) contains one or more genes contributed by one or more isolated genomes. Bars in the 14 layers

indicate the occurrence of GCs in a given isolated genome, black bars indicate presence of GCs, whereas grey indicates absence. GCs are organised based on their distribution across genomes, and layers of genomes are organised based on their average nucleotide identity (ANI), using Euclidean distance and ward ordination. Heatmap indicates ANI clusters of *Mycoplasma* isolates, where red indicates higher similarity between genomes. The five barplots below the heatmap indicate singleton GCs (dark brown), redundancy (dark brown), completion (blue), GC-content (dark green), and length of genomes (grey). Functional annotation of GCs with KEGG and COG were visualised in outer layers with green and red. The layers are grey if no KEGG or COG annotation were found. Notations in the outer layer of co-clustering of novel genomes are visualised with different colours. Samples from Atlantic Salmon were abbreviated "AS", samples from Rainbow Trout were abbreviated "RT", and samples from Whitefish were abbreviated "WF". **B) Comparison of KEGG classifications of novel *Mycoplasma.*** Venn diagram of KEGG functions concatenated from GCs from a co-assembly of the novel *Mycoplasma* genomes. Venn diagrams also include the nearest relative to the novel *Mycoplasma* genomes, including *Mycoplasma mobile* and *Mycoplasma iowae*.

Fluorescence in situ hybridization (FISH) of gut epithelia in rainbow trout

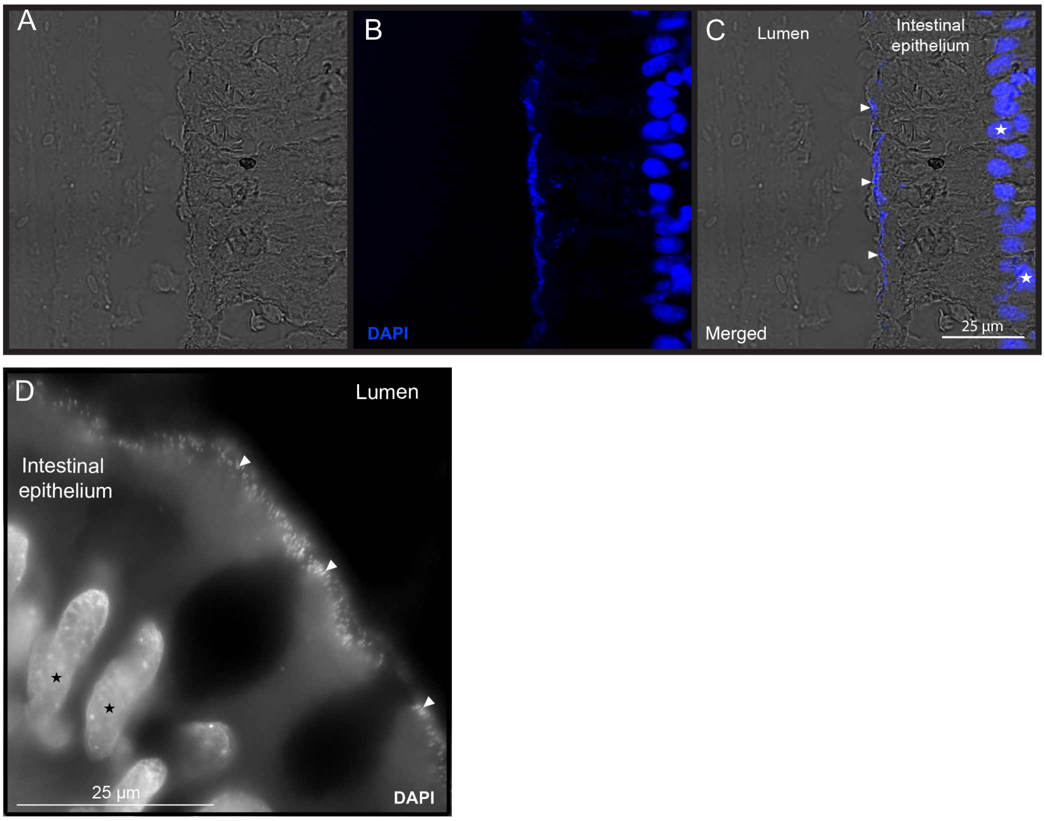

Supplementary Figure 4. Fluorescence imaging of gut epithelial in rainbow trout. Four µm rainbow trout intestine sections mounted with Vectashield with fluorescent DNA stain 4',6-diamidino-2-phenylindole (DAPI). Panel A, B and C are confocal images captured on a Leica SPX-5. The tissue was illuminated with A) white light, B) UV light and C) white light and UV light. In panel B and C DNA appears as a blue signal. Panel D was captured with Zeiss Axioplan fluorescence microscope and DNA is visible as white light. Stars represent nuclei of the fish tissue and arrow heads represent bacteria lining the intestinal wall.

Supporting phylogenomics of unknown *Mycoplasma* MAGs

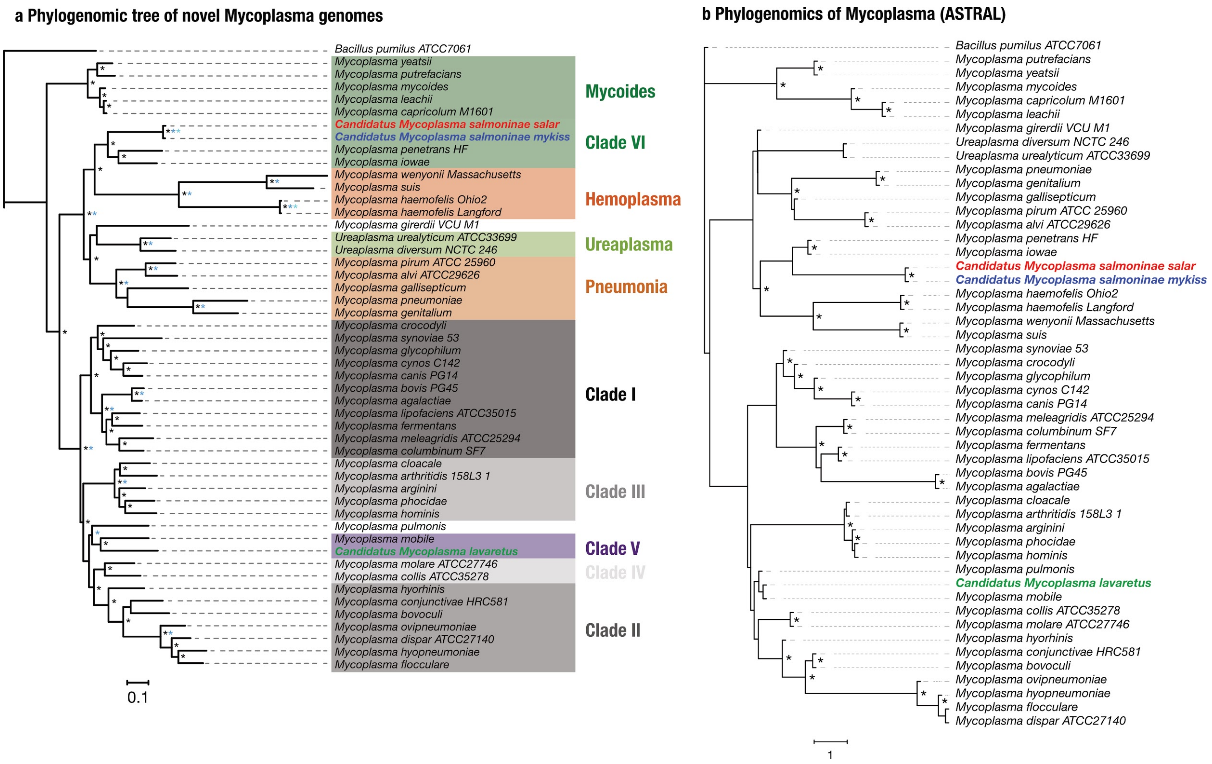

Supplementary Figure 5. Phylogenomics of unknown *Mycoplasma* MAGs. A. Phylogenomic tree inference from a concatenated alignment including 50 bacterial genomes (estimated in IQ-TREE). Asterisks indicate high branch supports (black: aLRT supports above 85; dark blue: gene internode certainty above 0.85; light blue: site internode certainty above 0.85). b. Phylogenomic tree estimated from ASTRAL, using the trees for core genes as input. Asterisks indicate branches with local posterior probabilities >0.85.

Metabolic reconstruction of intestinal related *Mycoplasma* species across multiple hosts

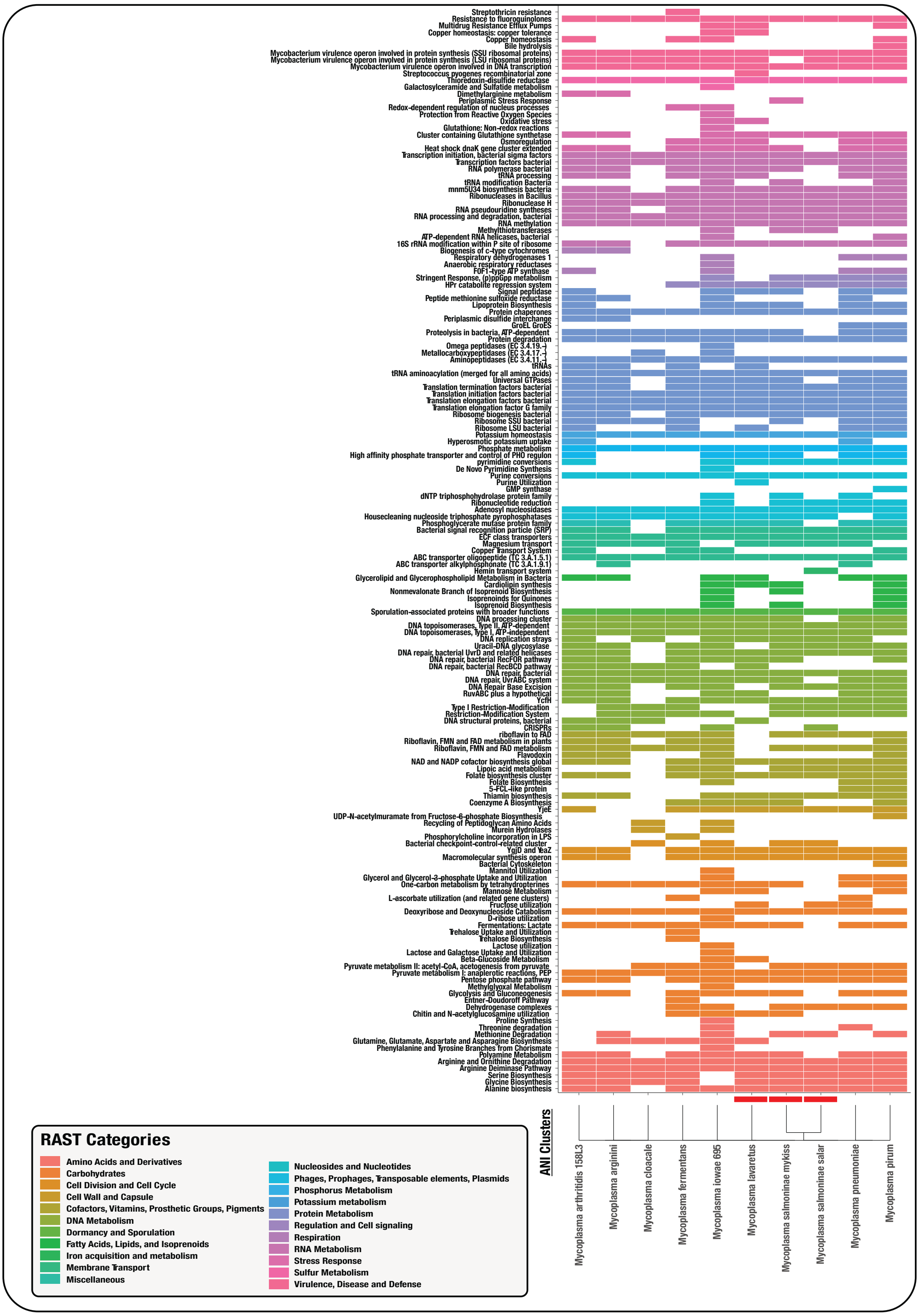

Supplementary Figure 6. Metabolic reconstruction of intestinal related *Mycoplasma* species across multiple known *Mycoplasma* clusters.

RAST subsystems are visualised for each *Mycoplasma* genome, including novel genomes. Visualisations are based on presence/absence of completed RAST subsystems. Colours in visualisation are based on RAST categories, which is shown in the legend. Arrangement of genomes are based on average nucleotide identity (ANI) and the dendrogram is based on hierarchical clustering with maximum likelihood of ward distances of ANI. Novel MAGs from salmonids are highlighted with a red bar on the x axis.

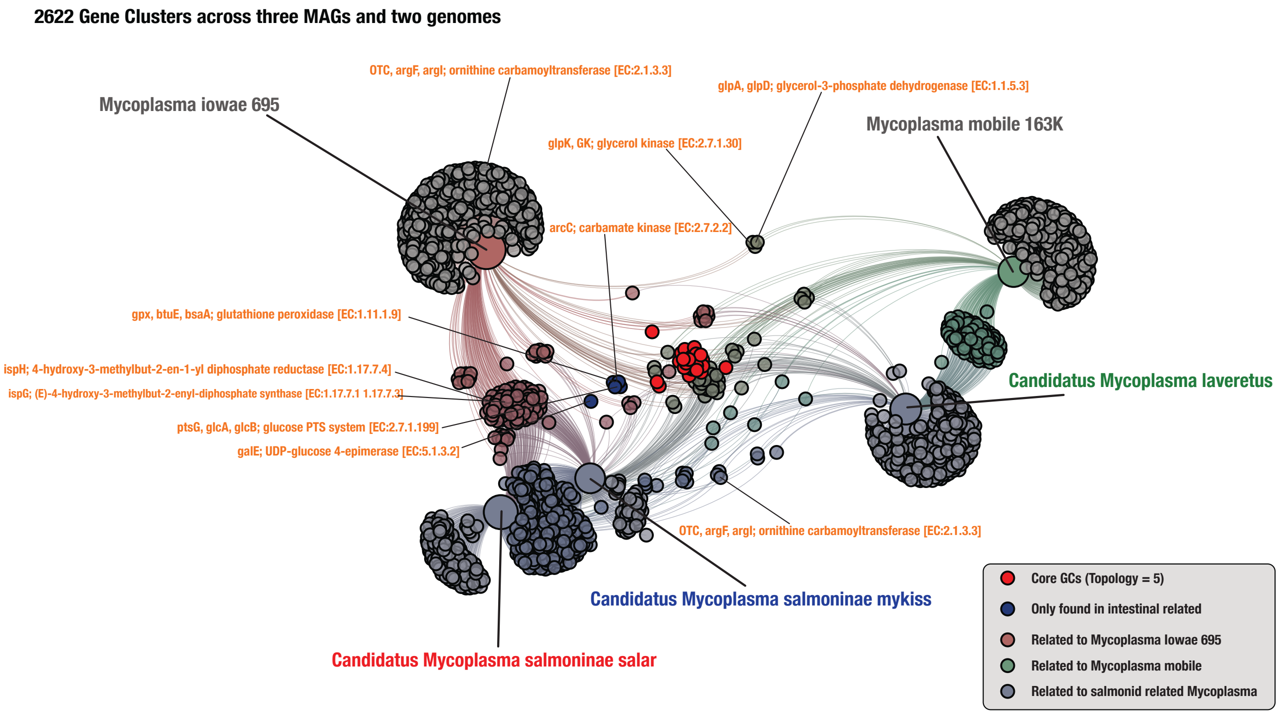

Supplementary Figure 7. Functional network of salmonid related *Mycoplasma* MAGs and their relatives. Functional network of the three MAGs and two genomes based on a total of 2622 identified gene clusters (GCs). Big nodes represent MAGs and genomes, whereas small nodes indicate GCs. Size and colour of genomic and MAG related nodes represent the number of related GCs and MAG taxonomy, respectively. Colours of functional nodes indicate their occurrence in the different MAGs and genomes. Name and KEGG accession number of functions of interest are highlighted in the figure.

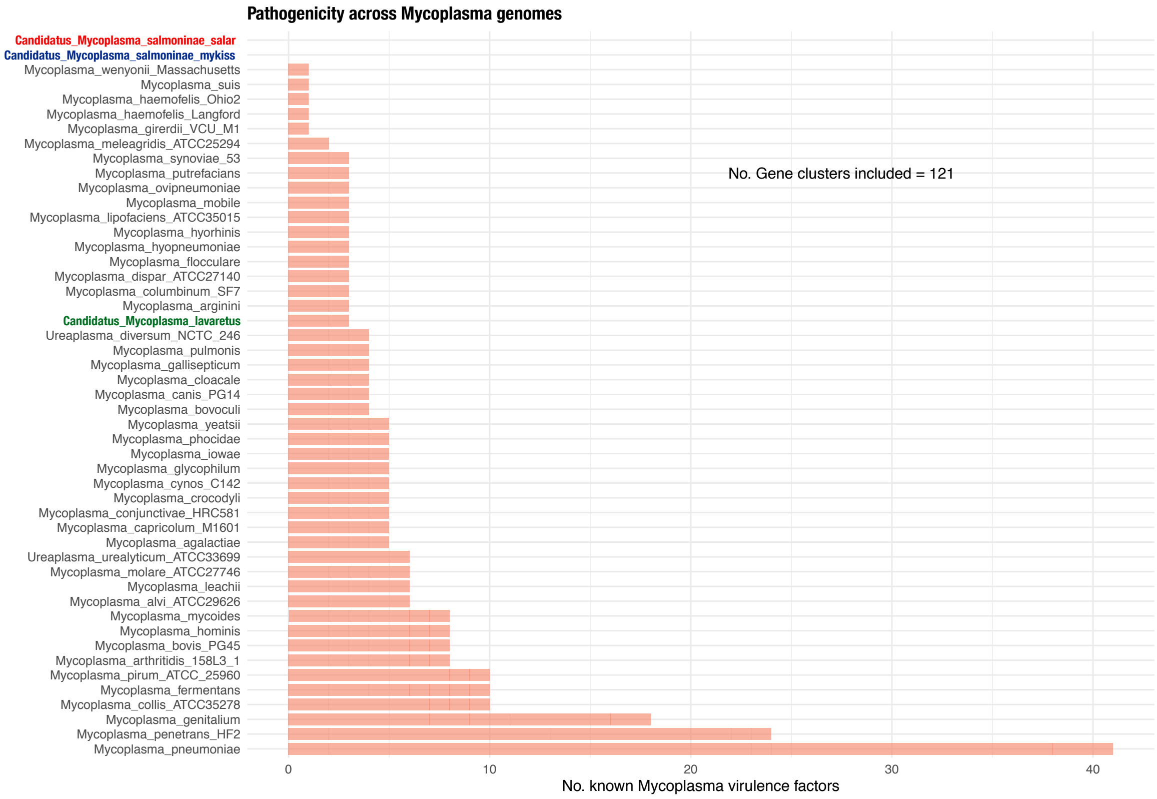

Supplementary Figure 8. Pathogenicity across *Mycoplasma* genomes based on known virulence factors present in *Mycoplasma*. Based on search for both functions, including virulence, toxins, anti-toxins, adhesion factors, Large Membrane Proteins (LMPs), and specific genes, including *traG/traE*, *glpF*, *oppA, mgpA/mgpC, katE,* across all gene clusters in the pangenome. Search were based on KEGG, COG, and Pfam.
