# Supplementary material for: Genome-resolved metagenomics suggests a mutualistic relationship between Mycoplasma and salmonid hosts
Source: Commun Biol. 2021 May 14;4:579. doi: 10.1038/s42003-021-02105-1 (PMC8121932; doi:10.1038/s42003-021-02105-1)
Supplement: Supplementary file 3 — Description of Supplementary Files [file 42003_2021_2105_MOESM3_ESM.pdf]

Description of Additional Supplementary Files

File name: Supplementary Data 1

Description: Summary of Mycoplasma genomes for comparative genomics.

File name: Supplementary Data 2

Description: Summary of RAST annotation of Mycoplasma genomes.

File name: Supplementary Data 3

Description: Summary of gene clusters in pangenome, including concatenated protein sequences.

File name: Supplementary Data 4

Description: Summary of Enrichment Analysis of KEGG annotations between intestinal and non-intestinal related Mycoplasma.
