# Supplementary material for: Genome-resolved metagenomics suggests a mutualistic relationship between Mycoplasma and salmonid hosts
Source: Commun Biol. 2021 May 14;4:579. doi: 10.1038/s42003-021-02105-1 (PMC8121932; doi:10.1038/s42003-021-02105-1)
Supplement: Supplementary file 8 — Reporting Summary [file 42003_2021_2105_MOESM8_ESM.pdf]

nature research

Corresponding author(s): Morten Tønsberg Limborg
Jacob Agerbo Rasmussen

Last updated by author(s): 2021.03.24

Reporting Summary

Nature Research wishes to improve the reproducibility of the work that we publish. This form provides structure for consistency and transparency in reporting. For further information on Nature Research policies, see our Editorial Policies and the Editorial Policy Checklist.

Statistics

For all statistical analyses, confirm that the following items are present in the figure legend, table legend, main text, or Methods section.

n/a	Confirmed	
☐	☒	The exact sample size (*n*) for each experimental group/condition, given as a discrete number and unit of measurement
☐	☒	A statement on whether measurements were taken from distinct samples or whether the same sample was measured repeatedly
☐	☒	The statistical test(s) used AND whether they are one- or two-sided<br>*Only common tests should be described solely by name; describe more complex techniques in the Methods section.*
☒	☐	A description of all covariates tested
☐	☒	A description of any assumptions or corrections, such as tests of normality and adjustment for multiple comparisons
☐	☒	A full description of the statistical parameters including central tendency (e.g. means) or other basic estimates (e.g. regression coefficient) AND variation (e.g. standard deviation) or associated estimates of uncertainty (e.g. confidence intervals)
☐	☒	For null hypothesis testing, the test statistic (e.g. *F*, *t*, *r*) with confidence intervals, effect sizes, degrees of freedom and *P* value noted<br>*Give P values as exact values whenever suitable.*
☒	☐	For Bayesian analysis, information on the choice of priors and Markov chain Monte Carlo settings
☐	☒	For hierarchical and complex designs, identification of the appropriate level for tests and full reporting of outcomes
☒	☐	Estimates of effect sizes (e.g. Cohen's *d*, Pearson's *r*), indicating how they were calculated

Our web collection on statistics for biologists contains articles on many of the points above.

Software and code

Policy information about availability of computer code

Data collection	External data information for comparative analysis can be found at: https://docs.google.com/spreadsheets/d/1m955W42GQtDrMuFvvdcQ1a-ssCFuuBDCSbYAR2FRUSo/edit?usp=sharing
Data analysis	Scripts for data analysis will be available, at https://github.com/JacobAgerbo/Comparative_Mycoplasma

For manuscripts utilizing custom algorithms or software that are central to the research but not yet described in published literature, software must be made available to editors and reviewers. We strongly encourage code deposition in a community repository (e.g. GitHub). See the Nature Research guidelines for submitting code & software for further information.

Data

Policy information about availability of data

All manuscripts must include a data availability statement. This statement should provide the following information, where applicable:
- Accession codes, unique identifiers, or web links for publicly available datasets
- A list of figures that have associated raw data
- A description of any restrictions on data availability

The novel Mycoplasma MAGs are located for review purpose in following share link: https://figshare.com/s/765447d5f1fba7ed3faf and will be publicly upon after acceptance at: 10.6084/m9.figshare.13019477.
Summary of each metagenome used to generate novel Mycoplasma MAGs are located for review purpose in following share link: https://figshare.com/s/a93fb5a38e6cef40961d and will be publicly available upon acceptance at: 10.6084/m9.figshare.13019507.
Summary of pangenome used to analyse novel Mycoplasma MAGs are located for review purpose in following share link: https://figshare.com/s/50483653045342877c8e and will be publicly available upon acceptance at: 10.6084/m9.figshare.13019543.
The raw host filtered dataset generated during the current study will be available in the ENA repository with study accession number PRJEB40990 acceptance of the

manuscript.

Field-specific reporting

Please select the one below that is the best fit for your research. If you are not sure, read the appropriate sections before making your selection.

☐ Life sciences ☐ Behavioural & social sciences ☒ Ecological, evolutionary & environmental sciences

For a reference copy of the document with all sections, see nature.com/documents/nr-reporting-summary-flat.pdf

Ecological, evolutionary & environmental sciences study design

All studies must disclose on these points even when the disclosure is negative.

Study description	For this comparative stusdy we carried out sampling of intestinal samples from three different salmonid host across scandinavia. Samples were aquired from different studies.
Research sample	Research samples were comprised from three different salmonids, including rainbow trout, Atlantic salmon, and European whitefish, which were sampled from different location across scandinavia. Genome resolving metagenomics were applied to resolve draft genomes of dominant salmonid related Mycoplasma. External genomes were aquired from GenBank to apply a comparative genomics study. External data information for comparative analysis can be found at: https://docs.google.com/spreadsheets/d/1m955W42GQtDrMuFvvdcQ1a-ssCFuuBDCSbYAR2FRUSo/edit?usp=sharing.
Sampling strategy	NA
Data collection	NA
Timing and spatial scale	Sampling of rainbow trout were carried out over 1 day, at 2019-04-23. Sampling of European whitefish were carried out over 1 day, at 2017-09-03. Sampling of Atlantic salmon were carried out over one day, at 2018-04-09.<br>All samples were preserved in standard preservation buffer, including SHIELD (Zymo Research) and 90% Ethanol, to minimize change in microbial composition.
Data exclusions	No data were excluded.
Reproducibility	NA
Randomization	This dataset is a subset of many samples, which were randomised prior to extraction of DNA.
Blinding	Blinding were not relevant for this comparative study, since this study is highly explorative.

Did the study involve field work? ☒ Yes ☐ No

Field work, collection and transport

Field conditions	Rainbow trout; Recirculated Aquatic System (RAS) based on freshwater, juvenile rainbow trout. Atlantic Salmon; In an open fjord net near Bergen for full grown salmon in marine water. European whitefish; freshwater lake, wild.
Location	Rainbow trout; BioMar Research facility (Hirtshals, Denmark). Atlantic Salmon; Lerøy production site in an Bergen, Coordinates; 60.499694, 4.928167. European whitefish; Lake Suohpatjavri.
Access & import/export	The methods for rainbow trout were performed in accordance with relevant guidelines and regulations and approved by The Danish Animal Experiments Inspectorate, under license no. 2015-15-0201-00645. The study is thus approved under the Danish law regarding experimental animals. Atlantic salmon and European whitefish included in this study were sacrificed immediately upon catch with a solid hit to the neck region resulting in instant death before tissue and gut content samples were taken. While no particular license is required for such sampling, we stress that all fish handling was supervised by experienced and trained staff in accordance with normal and legal procedures in Norway. We obtained permission for gill net fishing in Lake Suohpatjavri from the County Governor of Finnmark with legal authority through LOV 1992-05-15 nr 47, 113. Fish were euthanized by means of cerebral concussion prior to sample collection. No ethical permission is required from the Norwegian Animal Research Authority for collection with gill nets and the associated sacrifice of fish (FOR 1996-01-15 nr 23, the Norwegian Ministry of Agriculture and Food).
Disturbance	No disturbance were caused by the study.

Reporting for specific materials, systems and methods

We require information from authors about some types of materials, experimental systems and methods used in many studies. Here, indicate whether each material, system or method listed is relevant to your study. If you are not sure if a list item applies to your research, read the appropriate section before selecting a response.

Materials & experimental systems

n/a	Involved in the study	
☒	☐	Antibodies
☒	☐	Eukaryotic cell lines
☒	☐	Palaeontology and archaeology
☐	☒	Animals and other organisms
☒	☐	Human research participants
☒	☐	Clinical data
☒	☐	Dual use research of concern

Methods

n/a	Involved in the study	
☒	☐	ChIP-seq
☒	☐	Flow cytometry
☒	☐	MRI-based neuroimaging

Animals and other organisms

Policy information about studies involving animals; ARRIVE guidelines recommended for reporting animal research

Laboratory animals	Rainbow trout eggs were acquired from AquaSearch Ova (AquaSearch FRESH, Billund, Denmark, 100% females). Following disinfection with Desamar K30 (Foodtech AG, Uster, Schweiz) according to the manufacturer's instructions, the eggs were hatched and reared under pathogen-free conditions at the Bornholm Salmon Hatchery (Nexø, Denmark). During the rearing period, the fish were fed commercial pelleted feed. Rainbow trouts were reared on BioMar Research facility (Hirtshals, Denmark). In brief, all tanks were supplied through a closed recirculating water system. The water temperature was 14–15 °C, and filtering was performed through physical filtering, as well as a biofilter. The water was oxygenated and passed through an UV-filter (595 µJ/cm2). Sampling were perfomed 2019-04-23. Intestinal samples were preserved in preservation buffer (SHIELD) from Zymo Research.
Wild animals	European whitefish were aquired from a freshwater lake, Suohpatjavri , in northern norway. Sampling were carried out in the profundal zone. Whitefish were sampled 2017-09-03.<br>We obtained permission for gill net fishing in Lake Suohpatjavri from the County Governor of Finnmark with legal authority through LOV 1992-05-15 nr 47, 113. Fish were euthanized by means of cerebral concussion prior to sample collection. No ethical permission is required from the Norwegian Animal Research Authority for collection with gill nets and the associated sacrifice of fish (FOR 1996-01-15 nr 23, the Norwegian Ministry of Agriculture and Food). Intestinal samples were preserved in 90% Ethanol.
Field-collected samples	Sample collections for Atlantic salmon were taken from a Lerøy production site in a fjord near Bergen, Norway. Geographical Coordinates; 60.499694, 4.928167. Intestinal samples were preserved in preservation buffer (SHIELD) from Zymo Research. Samples were taken 2018-04-09.
Ethics oversight	The methods for rainbow trout were performed in accordance with relevant guidelines and regulations and approved by The Danish Animal Experiments Inspectorate, under license no. 2015-15-0201-00645. The study is thus approved under the Danish law regarding experimental animals. Atlantic salmon and European whitefish included in this study was sacrificed immediately upon catch with a solid hit to the head resulting in instant death before tissue and gut content samples were taken. While no particular license is required for such sampling, we stress that all fish handling was supervised by experienced and trained staff in accordance with normal and legal procedures in Norway.

Note that full information on the approval of the study protocol must also be provided in the manuscript.
